# Tumors and Cytomegalovirus: An Intimate Interplay

**DOI:** 10.3390/v14040812

**Published:** 2022-04-14

**Authors:** Georges Herbein

**Affiliations:** 1 Department Pathogens & Inflammation-EPILAB EA4266, University of Franche-Comté UBFC, 25000 Besançon, France; georges.herbein@univ-fcomte.fr; Tel.: +33-381-665-616; 2 Department of Virology, CHRU de Besançon, 25000 Besançon, France

**Keywords:** HCMV, oncomodulation, oncogenesis, PGCC, polyploid giant cancer cells, immunotherapy

## Abstract

Human cytomegalovirus (HCMV) is a herpesvirus that alternates lytic and latent infection, infecting between 40 and 95% of the population worldwide, usually without symptoms. During its lytic cycle, HCMV can result in fever, asthenia, and, in some cases, can lead to severe symptoms such as hepatitis, pneumonitis, meningitis, retinitis, and severe cytomegalovirus disease, especially in immunocompromised individuals. Usually, the host immune response keeps the virus in a latent stage, although HCMV can reactivate in an inflammatory context, which could result in sequential lytic/latent viral cycles during the lifetime and thereby participate in the HCMV genomic diversity in humans and the high level of HCMV intrahost genomic variability. The oncomodulatory role of HCMV has been reported, where the virus will favor the development and spread of cancerous cells. Recently, an oncogenic role of HCMV has been highlighted in which the virus will directly transform primary cells and might therefore be defined as the eighth human oncovirus. In light of these new findings, it is critical to understand the role of the immune landscape, including the tumor microenvironment present in HCMV-harboring tumors. Finally, the oncomodulatory/oncogenic potential of HCMV could lead to the development of novel adapted therapeutic approaches against HCMV, especially since immunotherapy has revolutionized cancer therapeutic strategies and new therapeutic approaches are actively needed, particularly to fight tumors of poor prognosis.

## 1. Introduction

Human cytomegalovirus (HCMV) or human herpesvirus 5 (HHV-5), one of the eight human herpesviruses, can establish lifelong latency within its corresponding host, as well as possessing reactivation potential. An exquisite equilibrium between the immunocompetent host and the virus itself allows viral presence for a lifetime parallel to improved viral fitness over time. Besides birth defects and congenital infections in the adult host, HCMV can cause symptoms due to its reactivation in immunosuppressed patients (organ transplant recipients) and immunocompromised patients (septic patients, elderly, HIV-infected patients, etc.) and favor age-related diseases such as atherosclerosis, immunosenescence, and malignancies [1,2]. Recent investigations have reported the presence of HCMV proteins and DNA in tumoral tissues of glioblastoma, neuroblastoma, colon cancer, breast cancer, ovarian cancer, and prostate adenocarcinoma [3,4,5,6,7]. Although some investigators fail to detect HCMV proteins and/or DNA in tumoral tissue, this might be linked to some technical limitations. Thus, the detection of HCMV proteins in tumor tissues is not just a random rare finding. Optimized PCR and immunohistochemistry protocols detect HCMV proteins and DNA in a majority of tissue specimens from a variety of tumors [8,9].

So far, seven human oncogenic viruses have been listed: Epstein–Barr virus (EBV), Kaposi sarcoma human virus (KSHV), human papillomavirus (HPV), hepatitis B and C viruses (HBV, HCV), human T-lymphotropic virus-1 (HTLV-1), and the most recently discovered Merkel cell polyomavirus (MCPyV) in January 2008. Although HCMV is not yet included in the oncogenic viruses list, it becomes more and more likely that HCMV is the eighth human oncovirus [6,10]. Although Koch’s postulation for viruses and cancer and Hill’s criteria for causation might be useful, they also have a limited understanding of virus-induced oncogenesis. Among the seven oncoviruses recognized so far, we can discriminate between direct tumor viruses (HPV, KSHV, HTLV1, EBV, HBV, and MCPyV) that either encode viral oncoproteins or activate host oncoproteins and indirect oncoviruses such as HCV, which set the stage for neoplasm mainly by chronic inflammation. This subclassification of oncoviruses might be artificial since some oncoviruses such as HBV produce a viral oncoprotein (HBx) parallel to inflammation and/or immune activation. The definitive classification of HCMV as the eighth oncovirus could be greatly facilitated by the discovery of one or several viral oncoproteins (e.g., such as E6 and E7 for HPV).

HCMV contributes to carcinogenesis as an initiator or promoter [11,12,13]. HCMV allows the tumor to escape immune surveillance by encoding viral proteins and inducing various immunosuppressive cellular factors allowing HCMV-induced immune tolerance, which favors tumor growth. In return, HCMV will increase its fitness in the immunologically weak environment of the cancerous cells [14]. Recently, HCMV has been reported to trigger the generation of polyploid giant cancer cells (PGCCs), as already reported for all human oncoviruses discovered so far [10,15]. PGCCs are keystone cancer cells resulting in stemness, chemoresistance, metastasis, and relapse in poor-prognosis cancers [16]. We and others have suggested revisiting the hallmarks of cancer to add a new hallmark, namely PGCCs and giant cell cycling [10,17,18].

Most of the oncoviruses, and especially oncoviruses, of the Herpesviridae family, namely EBV and KSHV, alternate lytic and latent phases to favor tumor initiation and spread [19]. Similarly, HCMV infection starts with viremia and lytic cycle, followed by latency in healthy individuals. Nevertheless, the homeostatic equilibrium established between HCMV and the host could be broken time by time by immunosuppressive conditions, during acute infections (bacterial and viral), transplantation, and even in physiological immune suppression such as in pregnancy. Immune imbalance in such immunocompromised patients can result in unhindered viral replication, followed by the reactivation of the latent virus in an inflammatory context [20], which will result in subsequent increased morbidity, but also, when resolved could favor HCMV fitness [21,22]. In fact, during a lifetime period, HCMV, through these successive periods of reactivation and latency, will genetically and phenotypically evolve to adapt to a moving immune landscape [23,24,25,26]. Various techniques, including restriction fragment length polymorphism (RLFP) analysis [27], targeted amplicon sequencing [28,29,30,31], and whole-genome sequencing [24,32], have been used to describe HCMV genome variability. With the increasing sequencing depth of next-generation sequencing platforms, the detection of low-frequency variants, i.e., minors, became possible [33]. Currently, there is mounting evidence that HCMV exists as a heterogeneous collection of genomes with variations in composition and distribution between anatomical compartments [34,35] and over time [24,32,33,35]. Performing high-throughput analysis, HCMV genome diversity is significantly more divergent than all other human herpesviruses and highlights the capacity of the viral genome to adapt to its host environment with high flexibility [36]. In addition to the high diversity of HCMV clinical strains [37,38], a key element to discriminate between oncogenic and oncomodulatory properties of HCMV strains is the limited potential of HCMV to replicate in already transformed cells [39]. Thus, HCMV is unable to productively infect most cancer-derived cell lines due to the fact that oncogenic alleles induce multiple restrictions to HCMV replication [39]. In contrast, the HCMV-DB and BL clinical strains isolated in our laboratory, which display oncogenic capacities in human epithelial mammary cells (HMECs), can fully replicate in primary epithelial cells with the alternation of both lytic and latent viral cycles and the appearance of CMV-transformed HMECs (CTH cells) up to several months post-infection [15,40]. Thus, a specific fitness for long-run epithelial cell replication of the HCMV oncogenic strains has to be taken into account to highlight the oncogenic properties of the virus.

During these prolonged periods of latency, numerous viral proteins are at play. For example, in endothelial cells, the UL133-UL138 locus, encoded in the ULb′ region of the HCMV genome, is essential for the viral late-stage response [41]. In infected cells, *UL135* and *UL136* genes promote viral maturation, but most importantly, this locus involves the main molecular switch among latency and reactivation, including the opposing roles of *UL135* and *UL138* (reviewed in [42]). The immune landscape is also essential to shape the outcome of antiviral immunity and is influenced by numerous viral determinants, including HCMV strain, virulence, MHC I downregulation, and other escape strategies elicited by HCMV during the early virus–host interaction [2]. This review highlights the considerable influence of HCMV on the immune landscape, the HCMV oncomodulatory and newly reported oncogenic signals that contribute to cancers, especially of poor prognosis.

## 2. HCMV and Tumor, a “Win–Win” Strategy

**Figure 1:**
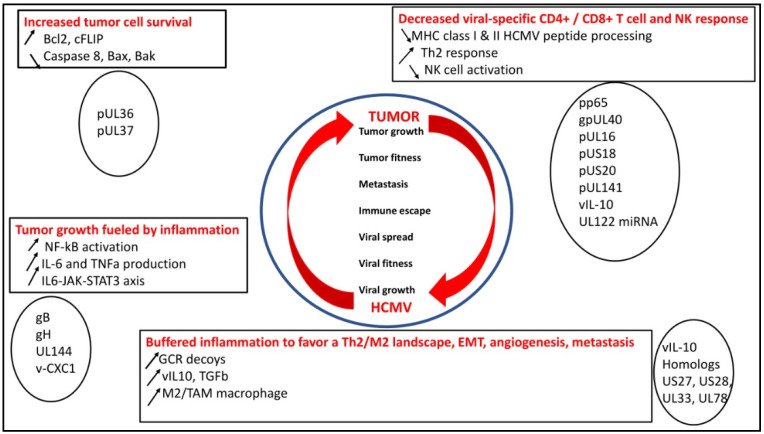
HCMV rewires the immune landscape to favor both HCMV fitness and tumor growth through several mechanisms: increased tumor cell survival, tumor growth fueled by inflammation parallel to the appearance of an M2/Th2 immune landscape, and escape from immune surveillance by decreased specific CD4^+^/CD8^+^ T-cell and NK responses.

HCMV, through its oncomodulatory effect, catalyzes the oncogenic process within the cancerous cells by expressing viral proteins known to possess a pro-oncogenic power such as IE1 and US28, among others, helping tumor cells to evade the immune system, preventing cell death, and favoring cell survival [6]. In addition, the restricted cellular immune responses against HCMV in these immune-privileged tumor sites will enhance HCMV replication in a context of constrained replication, as reported previously in HCMV infection of transformed cells [39]. On the other hand, cancer cells on their own escape immune control favoring epithelial–mesenchymal transition (EMT), metastasis, and relapse. Thus, the combination of the intrinsic cellular machinery and the viral immune escape strategies in cancer cells may offer an environment that enhances limited HCMV replication and boosts cancer cells to evade immune surveillance showing the bidirectional relationship between tumor cells and HCMV (Figure 1) [4,14,43].

### 2.1. HCMV Decreases Viral-Specific CD4^+^ and CD8^+^ T-Cell Response, NK Activity, which Could Favor Tumor Growth

HCMV fitness is a key factor for the virus to modulate the immune landscape. Thus, hindering the MHC class I-restricted antigen presentation is a key mechanism to favor long-lasting low replication of the virus [44]. The matrix protein, pp65, through its kinase activity, phosphorylates the IE1 protein and specifically inhibits the presentation of IE-derived antigenic peptides to escape immune recognition of the early produced viral proteins [45]. Knowing that pp65 is delivered directly into the cells during the viral fusion phase, HCMV will immediately escape from immunological surveillance [46]. In addition, pp65, which triggers the host interferon responses, inhibits NK cell detection or activation [47] and limits the recognition of CD4^+^ and CD8^+^ T cells by preventing MHC class I and II antigen processing and appearance [46]. Finally, a viral IL-10 (v-IL-10) homolog is produced in infected cells, which will create a Th2 immune landscape and counteract the anti-HCMV CD4^+^ and CD8^+^ T-cell responses [2].

To counteract the NK cell response parallel to the limited MHC class I expression observed in the vicinity of HCMV-infected cells, HCMV will display numerous molecular mechanisms [48]. gpUL40 and vIL10, respectively, increase the expression of HLA-E and HLA-G on the cell surface of infected cells [49,50,51]. The UL16 protein inhibits natural killer group 2D (NKG2D)-mediated NK cell activation by blocking the binding of NKG2D to UL16-binding proteins (ULBPs), namely ULBP1 and ULBP2, and to the MHC class I chain-related gene B (MICB gene) [52]. US18 and US20 viral proteins increase the destruction of MHC class I polypeptide-related sequence A (MICA), thereby limiting the NK cell from recognizing stress signals elicited by HCMV-infected cells [53]. To escape NK cell control, HCMV blocks NK cell-activating receptor (NKp30) by pp65, MICB gene expression by UL122-encoded microRNA, and CD155 expression by UL141 [52,54].

In contrast to healthy individuals with high and sustained HCMV-specific CTLs, in chronic HCMV infection, T-cell exhaustion occurs similar to the T-cell exhaustion observed in cancer patients [55]. Impaired immunological synapse formation and vesicle trafficking, loss of proliferative capacity, limited cytotoxicity, and decreased cytokine production by effector CD8^+^ T cells accompany T-cell exhaustion [56]. In addition, in HCMV-infected patients, the appearance of CMV-specific CD4^+^CD27^−^CD28^−^ T cells, which function as regulatory T cells (Treg), may explain their deleterious effects in CMV-infected individuals [57]. Additionally, CD28^−^ T cells have an inbuilt ability to release inflammatory cytokines and cytotoxic molecules that can damage tissues and amplify inflammatory pathways [58,59]. In addition, CMV infection upregulates Th17 cell activity during acute organ rejection with enhanced proinflammatory cytokine production [60]. Th17 cells exemplify immune adaptation. On the one hand, Th17 cells can have a full effector function protecting the host from pathogens with the associated risk of developing immune-mediated inflammatory diseases with enhanced proinflammatory cytokine production [61]. Th17 cells can also acquire an anti-inflammatory fate characterized by the secretion of the anti-inflammatory cytokine IL-10 [62]. Altogether, the increase in Treg, CD28^−^ T cells, and Th17 cells in chronic HCMV infection could favor tumor relapse and spread in cancer patients parallel to chronic inflammation [63]. Altogether, the impairment of HCMV-specific CD4^+^ and CD8^+^ T cells and NK cells function, parallel to the enhancement of the activity of Treg, CD28^−^ T cells, and Th17 cells could favor both viral spread and tumor development.

### 2.2. HCMV Favors Tumor Cell Survival

To favor cell survival and promote its own fitness, HCMV blocks the apoptotic machinery within infected cells. Thus, the expression of B-cell lymphoma 2 (Bcl-2) and Fas-associated death-domain-like IL-1β-converting enzyme-inhibitory proteins (FLIP) is upregulated in HCMV-infected cells, especially through IE2 [64,65]. pUL36 blocks the activity of procaspase 8 to the death-inducing signaling complex (DISC). pUL37 limits the action of proapoptotic Bcl-2 members, namely Bcl-2-associated X Protein (Bax) and Bcl-2 homologous antagonist/killer (*Bak*) [14,46]. Furthermore, HCMV has developed UL36 and UL37 proteins, which enhance the survival of infected cells, thus stimulating viral dissemination within the host [2]. The upregulation of the host-encoded complement regulatory proteins (CRPs) [66] and the integration of host cell-derived CRPs, CD55, and CD59 in virions curtail the complement attack and favor HCMV spread [14].

### 2.3. HCMV Buffers the Inflammatory Landscape and Favors a TH2/M2 Landscape

In addition, to counteract the cellular immune defense mediated through HCMV-specific CTLs and NK cells, HCMV constrains the inflammatory environment present in the vicinity of infected cells. Thus, upon viral entry, HCMV triggers the release of type I interferon (IFN), upregulates CD80 and CD86, and activates the NF-κB pathway leading to the production of inflammatory cytokines interleukin-6 (IL-6) and tumor necrosis factor-alpha (TNF-α) through the interaction of gB and gH with the toll-like receptor 2 (TLR2) [20,21,67]. In addition, UL144, a viral homolog of the TNF receptor and an IL-8-like chemokine (viral CXC-1), which favors the chemotaxis of peripheral blood neutrophils, will further participate in the proinflammatory microenvironment [68]. Such a proinflammatory microenvironment, although it might stimulate viral clearance through the activation of phagocytic cells [69], could also favor cellular transformation since the activation of the IL6/JAK/STAT3 axis could favor the appearance and the development of hepatocellular carcinoma (HCC), breast, and ovarian cancers [15,70,71,72]. In addition, the proinflammatory environment triggered by HCMV will favor a lytic cycle through several mechanisms. First, proinflammatory cytokines such as TNFa and IL-6 will activate the major immediate early promoter (MIEP) of HCMV, favor viral transcription, and reactivate the virus from latency. Second, a proinflammatory environment will shift the maturation of monocytes toward macrophages, the latter being much more permissive than the former for HCMV replication [73,74]. In addition, HCMV-infected macrophages can produce anti-inflammatory cytokines such as TGF-beta and IL-10 that will deactivate the T-cell present in the vicinity of infected M1 macrophages, but also might shift macrophages toward an M2 phenotype. Although HCMV susceptibility was higher in M2 macrophages, HCMV establishes a productive and persistent infection in both types of macrophages [75]. Thus, an exquisite balance between M1/M2 macrophage phenotype and HCMV replication kineticity could take place, especially with some HCMV strains, which are highly macrophage-tropic, such as the HCMV-DB strain [76].

HCMV might take advantage to buffer the proinflammatory microenvironment, diminish the antiviral immunity, and favor both viral spread and carcinogenesis. The production of G-protein-coupled receptors (GCRs) decoyed by HCMV, such as homologs US27, US28, UL33, and UL78, will hijack chemotactic factors, thus limiting the deleterious accumulation of the inflammatory cells at the viral infection site [77]. Thus, HCMV US28, a homolog of the CCR1 gene, binds CC chemokines RANTES, monocyte chemoattractant protein-1 (MCP-1), MCP3, macrophage inflammatory protein-1 alpha (MIP-1α), and MIP-1β, in addition to the membrane-associated CX3C chemokine, fractalkine [78]. Although US28 activates phospholipase C and NF-κB, its role in cellular transformation and viral dissemination makes it a dual viral protein present during both productive and latent viral infection [79,80]. In such a context of proinflammatory immune control by HCMV, the v-IL10 immunosuppressive cytokine limits the mitogen-stimulated peripheral blood mononuclear cells’ (PBMCs) proliferation and blocks proinflammatory cytokine synthesis in PBMCs and monocytes [81]. Of note, in such an immunocompromised microenvironment, HCMV enhances immune tolerance by inducing the transcription and release of TGF-β. TGF- β is well known to block antiviral IFN-γ and TNF-α cytokine production and cytotoxic effector activities of HCMV-specific Th1 cells and to be a key player in EMT, a critical event in the spread of transformed cells [2,82]. The majority of the HCMV-encoded proteins and microRNAs (miRNAs) involved in preventing immune recognition are active mostly during the lytic phase [79,80].

HCMV shapes a microenvironment in the vicinity of infected cells where immunosuppressive cytokines such as vIL-10 and TGFb could also pave the way for oncogenic transformation. In the presence of vIL-10, HCMV infection of human cancer stem cells triggers macrophage reprogramming to an M2 phenotype in the tumor microenvironment, leading to the appearance of macrophages close to tumor-associated macrophages (TAMs), a hallmark of poor prognosis in many cancers [83]. TAMs, the most abundant innate immune cells in tumors that exhibit an anti-inflammatory phenotype, are key players in cancer progression, metastasis, and resistance to therapy. A high TAM infiltration is generally associated with poor prognosis, but macrophages are highly plastic cells that can adopt either proinflammatory/antitumor or anti-inflammatory/protumor features in response to tumor microenvironment stimuli. Interestingly, upon CMV infection, macrophages undergo a morphological, immunophenotypic, and metabolic transformation process with features of stemness, altered migration, enhanced invasiveness, and provision of the cell cycle machinery for viral proliferation [84]. Thus, CMV profoundly perturbs macrophage identity beyond established limits of plasticity and rewires specific differentiation processes, allowing viral spread and impairing innate tissue immunity. Interestingly, we recently identified a highly macrophage-tropic HCMV strain (DB strain), that shifted the infected macrophages toward an M2/TAM phenotype and upon acute infection transformed primary human mammary epithelial cells, further linking some HCMV clinical strains with the M2/TAM phenotype and oncogenesis [6,71,76].

Angiogenesis is a key player in cancer expansion, tumor relapse, and metastasis. HCMV activates the STAT3 pathway and the vascular endothelial growth factor (VEGF), which will further favor angiogenesis [85,86]. vIL-10 and US28 will also enhance cancer cell invasion and metastasis [87]. The enhanced survival of neutrophils and mononuclear cells in the tumor microenvironment will further promote oncogenesis via the activation of an angiogenic switch [88,89]. Altogether, HCMV will promote a pro-oncogenic environment enabling cancer progression through the activation of cancer stem cells, angiogenesis, invasion, and an EMT phenotype [90,91].

## 3. HCMV Oncomodulation

**Figure 2:**
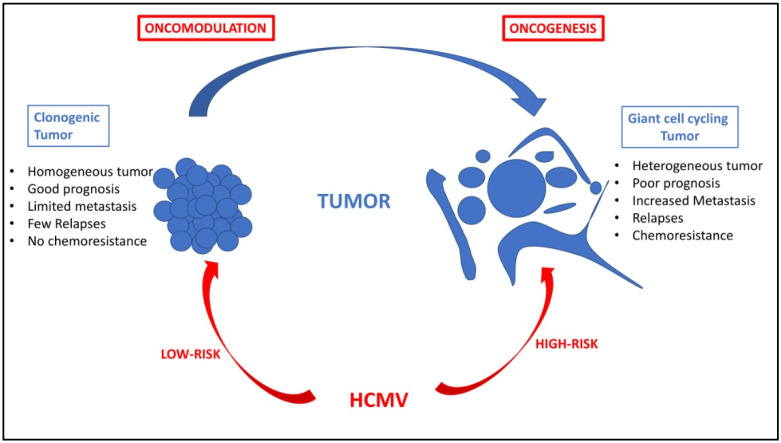
HCMV oncomodulation and oncogenesis: two paradigms for one tumor. Oncomodulation will result from the infection of tumor cells by HCMV strains (named low-risk strains), which will enhance the clonogenic development of the tumor and result in a tumor with quite controlled cell homogeneity, limited metastasis, few relapses, sensitivity to chemotherapy and radiotherapy, and an overall of good prognosis. In contrast, direct oncogenesis will result from the infection of primary cells by HCMV strains (named high-risk strains), which could participate in giant cell cycling with the appearance of polyploid giant cancer cells (PGCCs), in a tumor with highly heterogeneous cancer cells, increased risk of metastasis, more relapses, chemoresistance, and, ultimately, poor prognosis. Of note, the two paradigms, oncomodulation versus oncogenesis, might not be mutually exclusive but could coexist in the same patient depending on the fitness of the HCMV strain, which might vary over time.

The host immune system recognizes antigens resulting from alterations due to cancerous genetic and epigenetic instability [92,93], which can resist immune clearance by prompting tolerance in the presence of tumor-associated inflammatory cells [94]. Thus, the tumor microenvironment takes place in an immune landscape that could be impacted by HCMV. The first paradigm that emerges to explain the role of HCMV in cancer is named oncomodulation, where the virus enhances the oncogenesis process already occurring in the transformed cells. Thus, the oncomodulation paradigm relies on the indirect role of CMV in cancer (Figure 2). In fact, the prevalence of HCMV is remarkably high in several cancer forms [95], but it is very difficult to know if the presence of HCMV is incidental due to the viral infection on the top of an already present tumor in the context of an immunosuppressive TME, or if the HCMV by itself can initiate and promote the cancer program, and thereby could be defined as a genuine oncovirus.

HCMV proteins and DNA are detected in over 90% of breast, colon, and prostate cancer, rhabdomyosarcoma, hepatocellular cancer, salivary gland tumors, neuroblastoma, and brain tumors [95]. In addition, HCMV DNA was confirmed in up to 100% of breast cancer and 91% of sentinel lymph nodes samples from the metastatic group [96]. A potential link between HCMV and metastatic cancer has been suggested since HCMV is detected in 98% of breast cancer-derived metastatic brain tumors [97,98]. In metastatic breast cancer, the detection of HCMV results in shorter overall survival with the detection of HCMV DNA and transcripts in 92% and 80% of the used specimens, respectively [97]. A negative correlation has been reported between the detection of IE1 and hormone receptor expression, indicating a potential link between HCMV and hormone receptor-negative breast cancer tumors [71,99]. The development of HCMV-related proliferative diseases may partially be ascribed to the ability of US28 to activate cyclooxygenase-2 (COX-2) [78,100]. COX-2 is frequently expressed in many types of cancers, exerting a pleiotropic and multifaceted role in the genesis or promotion of carcinogenesis and cancer cell resistance to chemo- and radiotherapy [101]. Colon cancers, especially adenocarcinoma, often harbor high levels of HCMV, with IE1 and pp65 present in 82% and 78% of colorectal cancer samples with increased expression of Bcl-2 and COX-2 proteins, thus promoting colon cancer progression [102]. Hence, the use of COX-2-specific inhibitors, a subclass of nonsteroidal anti-inflammatory drugs (NSAIDs), and nonspecific COX-2 inhibitors such as aspirin reduce the cancer risk, potentiate antiangiogenic cancer therapy, and reduce metastasis in cancer patients [101,103]. The presence of HCMV was detected in preneoplastic and neoplastic prostate lesions, especially adenocarcinoma [104]. HCMV is also involved in disease progression in CNS tumors. Thus, HCMV is found in the majority of glioblastoma, and IE1 is expressed in glioma biopsy specimens in all grades [105]. In addition, IE and late-HCMV proteins are detected in 100% and 92% of primary neuroblastoma samples, respectively, including neuroblastoma cells, which express high levels of stemness markers such as CD133 and CD44 [106]. HCMV is also highly present in the primary medulloblastoma that expresses a high amount of IE and late-HCMV proteins, 92% and 73%, respectively [107]. Altogether, oncomodulation by HCMV has been observed in numerous adenocarcinoma and brain tumors.

## 4. HCMV Oncogenesis and the PGCC Paradigm

Besides the oncomodulation paradigm, a direct oncogenic effect of HCMV has been recently forwarded (Figure 2) [6]. First, among the multiple cell types infected by HCMV, the stem cells are permissive to HCMV, and stem cells markers such as Thy-1 and platelet-derived growth factor receptor alpha (PDGFRα) favor HCMV infection [108,109,110,111]. Since stem cells, especially cancer stem cells (CSCs), play a critical role in tumor initiation, cellular transformation, tumor heterogeneity, and cancer dissemination through EMT and metastasis, it is worth questioning the direct role of HCMV in oncogenesis, potentially through the infection of stem cells and/or the appearance of cancer stem cells. In addition, stem cells can lose control over their self-growth and renewal, act as a cancer source, and become susceptible to oncogenesis in the presence of inflammation and altered DNA repair pathways; the latter has been observed frequently with some HCMV strains [112,113]. HCMV could also favor stem cell survival, which would potentially sustain oncogenesis. Thus, IE1 protein promotes the presence of glioblastoma cancer stem cells through its induction of SRY-Box Transcription Factor 2 (SOX2), Nanog, Nestin, and octamer-binding transcription factor 4 (OCT3/4), key markers of stemness [114,115]. HCMV IE1 protein favors in glioblastoma cells the induction of transcription factors that are crucial for cancer stem cell persistence, cancer growth, and signaling pathways associated with the epithelial to mesenchymal (EMT) phenotype [114,116].

Tumors are renowned as intricate systems that harbor heterogeneous cancer cells with distinctly diverse molecular signatures, sizes, and genomic contents. Among those various genomic clonal populations within the complex tumoral architecture are the polyploid giant cancer cells (PGCCs). Although described for over a century, PGCCs are increasingly being recognized for their prominent role in tumorigenesis, metastasis, therapy resistance, and tumor repopulation after therapy. A shared characteristic among all tumors triggered by oncoviruses is the presence of polyploidy [10]. Recently, our research team has highlighted the role of PGCCs as a critical factor following infection with some strains of HCMV, which can be described as oncogenic (or high-risk) HCMV strains (in addition to the oncomodulatory effect of the virus) [6]. Two HCMV clinical strains isolated in our laboratory, named HCMV-DB and HCMV-BL, are capable of transforming primary human mammary epithelial cells (HMECs) into cytomegalovirus-transformed HMECs (CTH cells) and producing a “transcriptional profile” associated with DNA hypomethylation that results in the appearance of PGCCs in culture parallel to enhanced proliferation, activation of cancer stem cells, and EMT process [15,117,118]. In addition, IE1 expression was detected in CMV-transformed HMECs (CTH) cells, including PGCCs, which also express embryonic stem cell markers [15]. Xenografted NSG mice injected with CTH cells developed tumors that harbor HCMV DNA such as lncRNA4.9 gene, which displays a triple-negative basal-like phenotype [71]. Thus, following acute infection of mammary epithelial cells (HMECs) with some HCMV strains (DB, BL), our team has shown that transformed cells appear in culture including PGCCs and are tumorigenic in xenografted NSG mice, indicating the direct involvement of HCMV in oncogenesis. The molecular mechanisms involved in HCMV-induced oncogenesis are multifactorial, including, among others, cellular stress, polyploidy, and genomic instability parallel to stemness appearance [6,15]. HCMV gene products, including IE1 viral protein, could affect the pathways of p53 and Rb tumor suppressors and other pathways that are responsible for DNA repair parallel to increased expression of c-Myc in the transformed cells [11,119,120]. Presuming the role of HCMV gene products in causing DNA damage directly and indirectly, and stimulating proliferation in stem cells, HCMV (or at least some high-risk HCMV strains) has the potential to initiate and promote tumor formation, especially following acute infection of epithelial cells, which will result in the appearance of adenocarcinoma of poor prognosis such as triple-negative breast cancer.

Nonetheless, our data indicate that the oncogenic potency of HCMV clinical strains varies between low- and high-risk strains [15,40,71,118]. Only the high-risk HCMV strains can trigger the appearance of PGCCs [2,6]. HCMV-DB and HCMV-BL have been classified as high-risk strains since they showed a sustained transformation of acutely infected human mammary epithelial cells (HMECs) in vitro. These high-risk strains are characterized by elevated Myc expression, PI3K/Akt pathway activation, and p53 and Rb gene repression [15,71,121,122]. With regard to immune responses, Myc suppresses immune surveillance by modulating the expression of the innate immune regulator (CD47, also known as integrin-associated protein), the Treg activity, and the adaptive immune checkpoint, namely programmed death-ligand 1 (PD-L1) [123]. Further, PI3K/Akt hyperactivation observed with high-risk HCMV strains [15,71] induces immunosuppression and favors tumor initiation and progression via the activated Notch pathway [124]. Loss of Rb leads to an increase in IL-6 production recently involved in the appearance of PGCCs in ovarian cancer [125,126]. Thus, high-risk HCMV strains are potentially involved in the oncogenesis process as described previously [6,15,71] and might favor immune suppression in the tumor microenvironment further participating in tumor development. In addition, since the high-risk HCMV strains trigger the appearance of PGCCs, these latter are linked to undifferentiated heterogeneous tumors with poor prognosis; the immune environment of PGCCs needs definitively to be better characterized in the future.

**Figure 3:**
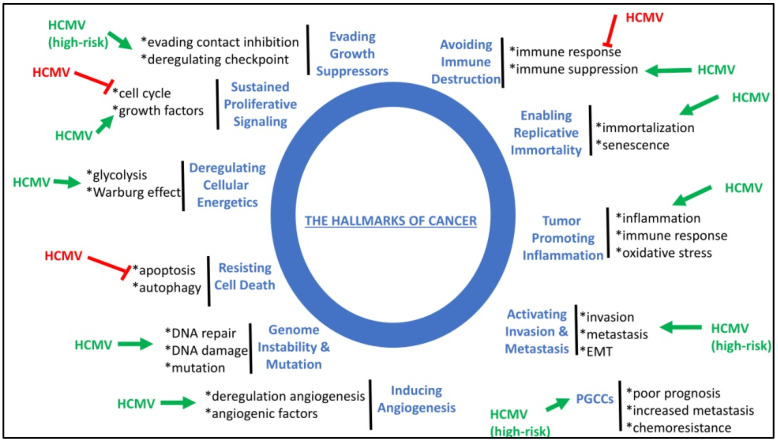
HCMV fulfills all the hallmarks of cancer and thereby could modulate the initiation and development of tumors. HCMV stimulates (green arrow) or blocks (red block) the molecular mechanisms involved in the hallmarks of cancer. The formation of PGCCs could be added as a new hallmark of cancer. High-risk HCMV strains might impact specifically some of the hallmarks of cancer where specified.

HCMV infection fulfills all the hallmarks of cancer, including sustained proliferative signaling, deregulating cellular energetics, resisting cell death, favoring genome instability and mutation, inducing angiogenesis, activating invasion and metastasis, tumor-promoting inflammation, enabling replicative immortality, and avoiding immune destruction (Figure 3) (reviewed in [127]). Although HCMV fulfills all the hallmarks of cancer, high-risk HCMV strains could specifically activate invasion and metastasis, evade growth suppressors and contact inhibition, and favor the appearance of PGCCs in tumors of poor prognosis (Figure 3). Thus, the presence of PGCCs, one of the features of oncoviruses (8), might be added as a new hallmark of cancer.

## 5. Antiviral Therapy and Immunotherapy Used to Fight HCMV-Linked Tumors

To confirm the role of HCMV in cancer development, glioblastoma (GBM) patients treated with valganciclovir, in addition to standard therapy, have demonstrated increased overall survival [127]. Patients with newly diagnosed glioblastoma, receiving valganciclovir, had a longer median overall survival, a higher 2-year survival rate, and a longer median time-to-tumor progression compared to controls [128]. Thus, valganciclovir prolonged the median overall survival of patients with newly diagnosed glioblastoma and was safe to use [128]. Although antiviral therapy has not been found to definitively improve outcomes for patients with GBM, several studies have leveraged CMV by targeting CMV antigens using ex vivo expanded T cells or dendritic cell vaccines with some promising results [129,130]. Thus, immunotherapy used to fight HCMV infection in cancer is under development. To boost the immune system of patients, adoptive T-cell therapy using CMV-specific T cells, or dendritic cells pulsed with CMV pp65 RNA to vaccinate GBM patients, has been assessed. Thus, GBM tumors were infiltrated by CMV-specific T cells, and GBM killing was enhanced by dendritic cells pulsed with a CMV-derived peptide from the viral pp65 via pp65-specific T cells, parallel to improved survival of the patients. Interestingly, unlike healthy donors, large proportions of CMV-specific T cells in GBM patients were dysfunctional [131]. Thus, immunotherapy based on vaccination and ex vivo T-cell expansion enhances the immune response against CMV-linked tumors and deserves further assessment (reviewed in [132,133]).

## 6. Conclusions

HCMV has been considered for decades as a herpesvirus involved mostly in asymptomatic or mild disease in immunocompetent individuals, which could lead to an important disease burden during congenital infection and in immunocompromised patients. Recently, the long-lasting effects of HCMV started to arise with its role in atherosclerosis, immunosenescence, and cancer. The oncomodulation paradigm could explain the accelerated progression of cancers when HCMV superinfection of the tumor occurs. Finally, very recently, a direct oncogenic effect of some HCMV strains, named high-risk strains, has been observed with cellular stress, appearance of PGCCs, stemness, and EMT, which could explain the appearance of aggressive cancers, especially adenocarcinoma, with poor prognosis, metastasis, and resistance to treatment. The oncomodulation and direct oncogenesis triggered by HCMV are not mutually exclusive and benefit from the same immunosuppressive tumor microenvironment. Overall, a better understanding of the complex role of HCMV in cancer and its immune landscape will lead to new therapeutic approaches that will curtail cancers, especially with poor prognosis.
